# Clinical Performance of the Loop-Mediated Isothermal Amplification Method Compared With Reverse Transcriptase Polymerase Chain Reaction for COVID-19 Diagnosis in Indonesia and the Philippines

**DOI:** 10.7759/cureus.106631

**Published:** 2026-04-08

**Authors:** Ryo Mizushima, Ivet M Suriapranata, Nelson Geraldino, Tri S Kurniasih, Febi Andriani, Cucunawangsih, Ratna S Wijaya, Nata P Lugito, Michele Hernandez-Diwa, Mark A Ang, Gabriel M Ozoa, Timothy Carl F Uy, Scarlett M Tabunar, Maria J Germar, Maria T Urbiztondo-Benedicto, Sho Saito, Kei Yamamoto, Masahiko Sakaguchi, Norimitsu Hosaka, Shota Koyano, Marlinang D Siburian, Maria R Umano-Urbiztondo, Takashi Kobayashi, Tetsuo Miura, Norio Ohmagari

**Affiliations:** 1 Disease Control and Prevention Center, National Center for Global Health and Medicine, Japan Institute for Health Security, Tokyo, JPN; 2 Division of Molecular Epidemiology, Mochtar Riady Institute for Nanotechnology, Universitas Pelita Harapan, Jakarta, IDN; 3 Department of Laboratories, Philippine General Hospital, University of the Philippines, Manila, PHL; 4 Department of Microbiology, Faculty of Medicine, Mochtar Riady Institute for Nanotechnology, Universitas Pelita Harapan, Jakarta, IDN; 5 Department of Internal Medicine, Faculty of Medicine, Mochtar Riady Institute for Nanotechnology, Universitas Pelita Harapan, Jakarta, IDN; 6 Department of Pathology, Philippine General Hospital, University of the Philippines, Manila, PHL; 7 Department of Emergency Medicine, Philippine General Hospital, University of the Philippines, Manila, PHL; 8 Department of Obstetrics and Gynecology, Philippine General Hospital, University of the Philippines, Manila, PHL; 9 Department of Radiology, Philippine General Hospital, University of the Philippines, Manila, PHL; 10 Department of Information and Communication Engineering, Osaka Electro-Communication University, Osaka, JPN; 11 Division of Research and Development, Biochemical Research Laboratory II, Eiken Chemical Co. Ltd., Tokyo, JPN; 12 Department of International Trials, Center for Clinical Sciences, Japan Institute for Health Security, Tokyo, JPN

**Keywords:** accuracy, ct value, indonesia, loop-mediated isothermal amplification, philippines, severe acute respiratory syndrome coronavirus 2

## Abstract

Real-time reverse transcriptase polymerase chain reaction (RT-PCR) is considered the gold standard for the diagnosis of coronavirus disease 2019 (COVID-19); however, this method is expensive, time-consuming, and requires skilled personnel. Therefore, this study aimed to evaluate the clinical performance of loop-mediated isothermal amplification (LAMP), a simpler test method.

A multinational prospective clinical performance study was conducted in Indonesia and the Philippines, comparing the LAMP method to RT-PCR for the diagnosis of COVID-19. In total, 256 subjects with COVID-19 symptoms underwent RT-PCR testing, of whom 130 and 126 were positive and negative, respectively.

In Indonesia (n = 180), 115 (63.9%) tested positive by RT-PCR and 65 (36.1%) tested negative; in the Philippines (n = 76), 15 subjects (19.7%) tested positive by RT-PCR and 61 (80.3%) tested negative. The LAMP and RT-PCR results were concordant in 243/256 subjects (94.9%; 95% confidence interval, 91.5-97.3) (92.8% in Indonesia, 100% in the Philippines). Among 130 RT-PCR-positive subjects, 117 were also positive by LAMP, yielding a sensitivity of 90%. Of the 126 RT-PCR-negative subjects, 126 were also negative by LAMP, yielding a specificity of 100%. The positive and negative predictive values were 100% and 90.6%, respectively.

Our study demonstrated high overall accuracy between the LAMP and RT-PCR methods, suggesting the potential utility of the LAMP method for detecting COVID-19 and highlighting its usefulness in managing cases.

## Introduction

Severe acute respiratory syndrome coronavirus 2 (SARS-CoV-2) infection (coronavirus disease 2019 (COVID-19)) was first reported in Wuhan, China, in December 2019 and rapidly spread, causing an unprecedented impact worldwide [1]. Southeast Asia is one of the regions greatly affected by this pandemic, with many cases reported in Indonesia and the Philippines. Both countries have large populations, with approximately 284.68 million and 116.34 million people, respectively [2]. As of December 20, 2023, the cumulative number of reported cases in Indonesia was 6,817,154, including 161,930 deaths [3], whereas the cumulative number of reported cases in the Philippines was 4,126,460, including 66,779 deaths [4].

Currently, real-time reverse transcriptase polymerase chain reaction (RT-PCR), which detects SARS-CoV-2 ribonucleic acid (RNA), is the gold standard method for diagnosis; however, RT-PCR is expensive, requires specialized technical skills, and has the disadvantage of taking a long time to obtain test results, making it difficult to introduce in some low- and middle-income countries [5]. Furthermore, before developing symptoms, people infected with COVID-19 may be asymptomatic but still excrete the virus; therefore, it is difficult to detect infection at this stage. As a result, when the pandemic began, strict measures, such as lockdowns, were implemented in many countries, significantly reducing the number of patients [6]. However, this resulted in significant socioeconomic damage, and, to maintain the economy, many countries reopened their borders, leading to the virus spreading again [7]. Therefore, an effective screening method that can easily, quickly, and cheaply detect the virus is essential to prevent further infection and an increase in mortality, especially in low- and middle-income countries, where medical and technical resources are limited.

Previous studies have shown that the loop-mediated isothermal amplification (LAMP) method is superior to standard RT-PCR in terms of simplicity and speed [8,9]. Furthermore, this testing method can be used with solar panels and batteries; therefore, it can also be used in low- and middle-income countries with underdeveloped power infrastructures. Therefore, this study aimed to evaluate the effectiveness of the LAMP method in detecting SARS-CoV-2 infection.

## Materials and methods

Study design and population

This prospective multinational clinical performance study was conducted in Indonesia and the Philippines from August 2021 to December 2022. The study was conducted at two facilities in Jakarta, Indonesia (Mochtar Riady Institute for Nanotechnology at (1) Siloam Hospitals Lippo Village/Rumah Sakit Umum Siloam Lippo Village and (2) Siloam Hospital Kelapa Dua), and one facility in Manila, Philippines (University of the Philippines, Philippine General Hospital).

The protocol, informed consent forms, recruitment materials, and all subject materials were reviewed and approved by the Ethical Review Committee of each site and the National Center for Global Health and Medicine in Japan before any subject was enrolled in the study (NCGM-G-004100-00). This study was conducted in accordance with the International Council for Harmonisation Good Clinical Practice guidelines. All subjects provided informed consent prior to study participation.

LAMP and PCR assays

Nasopharyngeal swabs were collected from all the subjects. The specimens were suspended in a virus transport medium (VTM) and transferred to the laboratory for RNA extraction using a QIAamp Viral RNA kit (QIAGEN, Hilden, Germany). In Indonesia, specimens were collected using a Virus Transport Kit (2 mL; Jinan Babio Tech, Jinan, China), whereas in the Philippines, Bioteke VTM (3 mL; BioTeke Corporation, Wuxi, China) was used.

The Loopamp™ SARS-CoV-2 Detection Kit (Eiken Chemical Co., Ltd., Tokyo, Japan) was used for the LAMP method, targeting the nucleocapsid (N) and RNA-dependent RNA polymerase genes of SARS-CoV-2. Testing was performed according to the manufacturer’s instructions. The reaction mixture was prepared by combining the RNA sample (10 μL) with the provided master mix (15 μL), containing the primer set, to a final volume of 25 μL. The mixture was incubated at 62.5°C for 35 min, inverted for 2 min, mixed five times, and spun down. Turbidity was measured using a real-time turbidimeter, the Loopamp EXIA (Eiken Chemical Co., Ltd.) [10,11].

For the RT-PCR method, the reaction was carried out at 50°C for 30 min and 95°C for 15 min, followed by cycling at 15 s at 95°C and 1 min at 60°C. Synthesized RNA that contained an artificial sequence was used as a positive control to identify laboratory contamination, whereas distilled water was used as a negative control. A result was considered positive if it occurred within 40 cycles [12]. The staff performing the LAMP and RT-PCR assays were not blinded. In addition, online training was provided to the staff who performed the LAMP method.

Inclusion and exclusion criteria

Subjects suspected of having a SARS-CoV-2 infection, who met all the inclusion criteria listed below and did not meet any of the exclusion criteria, were included in this study.

The inclusion criteria were as follows: subjects assessed by the investigator or sub-investigator of this study to have symptoms of SARS-CoV-2 infection based on the national guidelines for COVID-19 in each country [13,14]: a nasopharyngeal swab sample could be collected; written consent was voluntarily provided by the subject or legally represented to participate in the study; and aged 18 years or older (subjects over 18 years of age are regarded as adults in the Philippines and Indonesia and considered to have attained the legal age for consent to participate in the study).

The exclusion criteria were as follows: subjects with mucosal damage or nasopharyngeal lesions; nasopharyngeal pain, trauma, or nasal surgery within one month; a significantly deviated nasal septum; a history of chronically obstructed nasal passages; severe coagulation disorders; subjects suspected of using an illicit drug within the last 12 months; and subjects determined to be ineligible by the investigator.

Data collection and outcome measures

The collected demographic characteristics included patient age, sex, hospitalization/outpatient status, history of contact with infected individuals, and severity, as well as the number of days from symptom onset to the LAMP procedure. Disease severity was defined as follows: mild (SpO₂ ≥96%), moderate I (93% < SpO₂ < 96%), moderate II (SpO₂ ≤93%), and severe (requiring admission to the intensive care unit or mechanical ventilation). The primary endpoint was the accuracy of the LAMP results compared with RT-PCR. The secondary endpoints were the accuracy, sensitivity, and specificity of LAMP compared with RT-PCR methods, stratified by cycle threshold (Ct) value and the number of days from symptom onset to the LAMP procedure, as well as a comparison between the two countries.

Statistical methods

In this study, we evaluated the ability of the LAMP method to predict RT-PCR results by calculating the accuracy, sensitivity, specificity, positive predictive value (PPV), negative predictive value (NPV), and their corresponding Clopper-Pearson 95% confidence intervals (CIs). These performance metrics were stratified by country and by differences in Ct values. Statistical analyses were performed using SAS Enterprise Guide 8.2 (SAS Institute, Cary, NC, USA).

Sample size calculation

The specificity and sensitivity of the LAMP assay were assumed to be 99% and 95.7%, respectively. This was set by estimating them to be 1% lower than previous data [15], which had a specificity of 100%, while the sensitivity remained unchanged. To show that the LAMP method is equivalent to the RT-PCR method by setting the CI at 95% (specificity ± 2.5%, sensitivity ± 2.5%), the lower limit of specificity should be ≥96.5%, and the lower limit of sensitivity should be ≥93.2%. Assuming a 50% positive rate in the target population (suspected cases of COVID-19), 122 samples were required to meet these conditions for the sensitivity analysis, and 506 for the specificity analysis. Therefore, in theory, a sample size of 506 is required to cover both the sensitivity and specificity analyses. However, in response to the emergency situation, where the number of people infected with COVID-19 continued to increase, the “Instructions and Requirements for Emergency Use Listing (EUL) Submission” [16] by the World Health Organization (WHO) stipulates that in vitro diagnostic products that detect SARS-CoV-2 nucleic acid, and rapid diagnostic products that detect SARS-CoV-2 antigens, should be tested on at least 100 samples (lower or upper respiratory tract, depending on the sample category). In accordance with this regulation, and because recruitment of positive subjects was challenging due to the declining positivity rate at the tail end of the pandemic, this study aimed to obtain more than 100 positive and negative COVID-19 results, and recruitment was terminated when the target number was reached. Following the WHO guidance on EUL submission, the results interpretation focused on point estimates of the concordance, sensitivity, and specificity, rather than the specific limits of the CIs.

## Results

A total of 256 subjects were enrolled. The distribution of participants by country was 180 (70.3%) from Indonesia and 76 (29.7%) from the Philippines. Among the Indonesian and Philippine participants, 63.9% and 19.7%, respectively, were RT-PCR positive. Overall, 50.8% of participants were RT-PCR positive.

Primary endpoint

Of the 256 subjects, 243 (94.9%; 95% CI, 91.5-97.3) showed concordant results between the LAMP and RT-PCR methods (Table 1).

**Table 1:** Positive predictive value, negative predictive value, sensitivity, and specificity - overall (Indonesia and the Philippines) RT-PCR: real-time polymerase chain reaction; LAMP: loop-mediated isothermal amplification; CI: confidence interval

	RT-PCR-positive (n = 130)	RT-PCR-negative (n = 126)	Positive predictive value % (95% CI)	Negative predictive value % (95% CI)	Sensitivity % (95% CI)	Specificity % (95% CI)	Accuracy % (n) (95% CI)
LAMP-positive	117	0	100 (96.9-100)	90.6 (84.5-94.9)	90.0 (83.5-94.6)	100 (97.1-100)	94.9 (243/256) (91.5-97.3)
LAMP-negative	13	126					

Secondary endpoints

Of the 130 RT-PCR-positive subjects, 117 were also positive by LAMP (sensitivity, 90%; 83.5-94.6), and all 126 RT-PCR-negative subjects were also negative by LAMP (specificity, 100%; 97.1-100). The PPV and NPV were 100% (96.9-100) and 90.6% (84.5-94.9), respectively.

Table 2 shows the stratified analysis by Ct value (≤30, ≤32, ≤35, all) and by the number of days from symptom onset to the LAMP procedure (≤2 days, ≤3 days, ≤4 days, all). Accuracy decreased as the Ct value increased, from 100% (98.4-100) at ≤30, to 99.6% (97.6-100) at ≤32, 96.8% (93.7-98.6) at ≤35, and 94.9% (91.5-97.3) for all. A longer interval between symptom onset and the LAMP procedure was also associated with a decrease in accuracy (95.7%; 91.7-98.1 within two days, 95.2%; 91.6-97.6 within four days, and 94.9%; 91.5-97.3 for all), although the decline was minimal and much smaller than that observed for Ct value (Table 2).

**Table 2:** Stratified data for Ct value and days from symptom onset to the LAMP procedure - overall (Indonesia and the Philippines) RT-PCR: real-time polymerase chain reaction; LAMP: loop-mediated isothermal amplification; Ct: cycle threshold; CI: confidence interval

	RT-PCR-positive (n = 130)	RT-PCR-negative (n = 126)	Positive predictive value % (95% CI)	Negative predictive value % (95% CI)	Sensitivity % (95% CI)	Specificity % (95% CI)	Accuracy % (n) (95% CI)
Ct value							
≤30 (n = 224)			100 (97.1-100)	100 (96.3-100)	100 (96.3-100)	100 (97.1-100)	100.0 (224/224) (98.4-100)
LAMP-positive	98	0					
LAMP-negative	0	126					
≤32 (n = 230)			99.2 (95.7-100)	100 (96.5-100)	99.0 (94.8-100)	100 (97.1-100)	99.6 (229/230) (97.6-100)
LAMP-positive	103	0					
LAMP-negative	1	126					
≤35 (n = 247)			94.0 (88.6-97.4)	100 (96.8-100)	93.4 (87.4-97.1)	100 (97.1-100)	96.8 (239/247) (93.7-98.6)
LAMP-positive	113	0					
LAMP-negative	8	126					
All (n = 256)			90.6 (84.5-94.9)	100 (96.9-100)	90.0 (83.5-94.6)	100 (97.1-100)	94.9 (243/256) (91.5-97.3)
LAMP-positive	117	0					
LAMP-negative	13	126					
Days from symptom onset to LAMP procedure							
≤2 (n = 185)			92.0 (84.8-96.5)	100 (95.8-100)	91.4 (83.8-96.2)	100 (96.1-100)	95.7 (177/185) (91.7-98.1)
LAMP-positive	85	0					
LAMP-negative	8	92					
≤3 (n = 216)			91.7 (85.3-96.0)	100 (96.2-100)	90.5 (83.2-95.3)	100 (96.7-100)	95.4 (206/216) (91.7-97.8)
LAMP-positive	95	0					
LAMP-negative	10	111					
≤4 (n = 229)			91.4 (85.1-95.6)	100 (96.4-100)	90.2 (83.1-95.0)	100 (96.9-100)	95.2 (218/229) (91.6-97.6)
LAMP-positive	101	0					
LAMP-negative	11	117					
All (n = 256)			90.6 (84.5-94.9)	100 (96.9-100)	90.0 (83.5-94.6)	100 (97.1-100)	243/256 (94.9) (91.5-97.3)
LAMP-positive	117	0					
LAMP-negative	13	126					

Table 3 shows the differences in the demographic and baseline characteristics between Indonesia and the Philippines. In Indonesia, approximately half the participants (51.1%) were outpatients, whereas in the Philippines, almost all participants (94.7%) were outpatients. In Indonesia, 66 subjects (36.7%) had a history of contact with infected individuals, compared to 42 (55.3%) in the Philippines. Severity was predominantly mild in both Indonesia and the Philippines (87.2% and 86.8%, respectively). The median number of days from symptom onset to LAMP was two days (interquartile range (IQR): 1-3) in Indonesia and one day (IQR: 1-2) in the Philippines.

**Table 3:** Participant demographics and baseline characteristics IQR: interquartile range; LAMP: loop-mediated isothermal amplification

	Indonesia (n = 180)	The Philippines (n = 76)	Overall (n = 256)
Age (years), median (IQR)	31.5 (26-51)	31.5 (26-44)	31.5 (26-48)
Male, n (%)	78 (43.3)	22 (27.6)	99 (38.7)
Hospitalization/Outpatient, n (%)			
Hospitalization	88 (48.9)	4 (5.3)	92 (35.9)
Outpatient	92 (51.1)	72 (94.7)	164 (64.1)
History of Contact With Infected Person, n (%)	66 (36.7)	42 (55.3)	108 (42.2)
Severity			
Mild	157 (87.2)	66 (86.8)	223 (87.1)
Moderate I	5 (2.8)	10 (13.2)	15 (5.9)
Moderate II	6 (3.3)	0 (0)	6 (2.3)
Severe	12 (6.7)	0 (0)	12 (4.7)
Days from symptom onset to LAMP procedure (days), median (IQR)	2 (1-3)	1 (1-2)	1.5 (1-3)

In Indonesia, the accuracy between the LAMP and RT-PCR methods was 92.8% (88.0-96.1); the PPV of the LAMP method was 100% (96.4-100); the NPV was 83.3% (73.2-90.8); sensitivity was 88.7% (81.4-93.8); and specificity was 100% (94.5-100). In the Philippines, the accuracy was 100% (95.3-100). Accordingly, the PPV and NPV of the LAMP method were 100% (94.1-100) and 100% (78.2-100), respectively, and both the sensitivity and specificity were 100% (78.2-100) and 100% (94.1-100), respectively (Table 4).

**Table 4:** Positive predictive value, negative predictive value, sensitivity, and specificity - individual data for Indonesia and the Philippines RT-PCR: real-time polymerase chain reaction; LAMP: loop-mediated isothermal amplification; CI: confidence interval

Country		RT-PCR-positive	RT-PCR-negative	Positive predictive value % (95% CI)	Negative predictive value % (95% CI)	Sensitivity % (95% CI)	Specificity % (95% CI)	Accuracy % (n) (95% CI)
Indonesia	LAMP-positive	102	0	100 (96.4-100)	83.3 (73.2-90.8)	88.7 (81.4-93.8)	100 (94.5-100)	92.8 (167/180) (88.0-96.1)
	LAMP-negative	13	65					
The Philippines	LAMP-positive	15	0	100 (94.1-100)	100 (78.2-100)	100 (78.2-100)	100 (94.1-100)	100 (76/76) (95.3-100)
	LAMP-negative	0	61					

Tables 5-6 show the stratified data analysis based on the Ct value and the number of days from symptom onset to LAMP in Indonesia and the Philippines, respectively. In Indonesia (n = 180), the accuracy with the LAMP method for Ct values ≤30 (83.9%, n = 151), ≤32 (86.7%, n = 156), and ≤35 (96.1%, n = 173) was 100%, 99.4%, and 95.4%, respectively, which tended to be somewhat low. In addition, the accuracy for ≤2 (69.4%, n = 125), ≤3 (80%, n = 144), and ≤4 (86.7%, n = 156) days after symptom onset was 93.6% (87.8-97.2), 93.1% (87.6-96.6), and 92.9% (87.7-96.4), respectively, which tended to be somewhat low. We reviewed 13 Indonesian cases where LAMP and RT-PCR results did not match. The Ct values for these 13 cases ranged from 31 to 36, with a median (IQR) of 35 (34-36).

**Table 5:** Stratified data for Ct value and days from symptom onset to the LAMP procedure - Indonesia RT-PCR: real-time polymerase chain reaction; LAMP: loop-mediated isothermal amplification; Ct: cycle threshold; CI: confidence interval

	RT-PCR-positive (n = 115)	RT-PCR-negative (n = 65)	Positive predictive value % (95% CI)	Negative predictive value % (95% CI)	Sensitivity % (95% CI)	Specificity % (95% CI)	Accuracy % (n) (95% CI)
Ct value							
≤30 (n = 151)			100 (94.5-100)	100 (95.8-100)	100 (95.8-100)	100 (94.5-100)	100 (151/151) (97.6-100)
LAMP-positive	86	0					
LAMP-negative	0	65					
≤32 (n = 156)			98.5 (91.8-100)	100 (96.0-100)	98.9 (94.0-100)	100 (94.5-100)	99.4 (155/156) (96.5-100)
LAMP-positive	90	0					
LAMP-negative	1	65					
≤35 (n = 173)			89.0 (79.5-95.1)	100 (96.4-100)	92.6 (85.9-96.7)	100 (94.5-100)	95.4 (165/173) (91.1-98.0)
LAMP-positive	100	0					
LAMP-negative	8	65					
All (n = 180)			83.3 (73.2-90.8)	100 (96.4-100)	88.7 (81.4-93.8)	100 (96.4-100)	92.8 (167/180) (88.0-96.1)
LAMP-positive	102	0					
LAMP-negative	13	65					
Days from symptom onset to LAMP procedure							
≤2 (n = 125)			85.2 (72.9-93.4)	100 (94.9-100)	89.9 (81.0-95.5)	100 (92.3-100)	93.6 (117/125) (87.8-97.2)
LAMP-positive	71	0					
LAMP-negative	8	46					
≤3 (n = 144)			84.1 (72.7-92.1)	100 (95.5-100)	89.0 (80.7-94.6)	100 (93.3-100)	93.1 (134/144) (87.6-96.6)
LAMP-positive	81	0					
LAMP-negative	10	53					
≤4 (n = 156)			84.3 (73.6-91.9)	100 (95.8-100)	88.7 (80.6-94.2)	100 (93.9-100)	92.9 (145/156) (87.7-96.4)
LAMP-positive	86	0					
LAMP-negative	11	59					
All (n = 180)			83.3 (73.2-90.8)	100 (96.4-100)	88.7 (81.4-93.8)	100 (94.5-100)	92.8 (167/180) (88.0-96.1)
LAMP-positive	102	0					
LAMP-negative	13	65					

**Table 6:** Stratified data for Ct value and days from symptom onset to the LAMP procedure - The Philippines RT-PCR: real-time polymerase chain reaction; LAMP: loop-mediated isothermal amplification; Ct: cycle threshold; CI: confidence interval

	RT-PCR-positive (n = 15)	RT-PCR-negative (n = 61)	Positive predictive value % (95% CI)	Negative predictive value % (95% CI)	Sensitivity % (95% CI)	Specificity % (95% CI)	Accuracy % (n) (95% CI)
Ct value							
≤30 (n = 73)			100 (94.1-100)	100 (73.5-100)	100 (73.5-100)	100 (94.1-100)	100 (73/73) (95.1-100)
LAMP-positive	12	0					
LAMP-negative	0	61					
≤32 (n = 74)			100 (94.1-100)	100 (75.3-100)	100 (75.3-100)	100 (94.1-100)	100 (74/74) (95.1-100)
LAMP-positive	13	0					
LAMP-negative	0	61					
≤35 (n = 74)			100 (94.1-100)	100 (75.3-100)	100 (75.3-100)	100 (94.1-100)	100 (74/74) (95.1-100)
LAMP-positive	13	0					
LAMP-negative	0	61					
All (n = 76)			100 (94.1-100)	100 (78.2-100)	100 (78.2-100)	100 (94.1-100)	100 (76/76) (95.3-100)
LAMP-positive	15	0					
LAMP-negative	0	61					
Days from symptom onset to LAMP procedure							
≤2 (n = 60)			100 (92.3-100)	100 (76.8-100)	100 (76.8-100)	100 (92.3-100)	100 (60/60) (94.0-100)
LAMP-positive	14	0					
LAMP-negative	0	46					
≤3 (n = 72)			100 (93.8-100)	100 (76.8-100)	100 (76.8-100)	100 (93.8-100)	100 (72/72) (95.0-100)
LAMP-positive	14	0					
LAMP-negative	0	58					
≤4 (n = 73)			100 (93.8-100)	100 (78.2-100)	100 (78.2-100)	100 (93.8-100)	100 (73/73) (95.1-100)
LAMP-positive	15	0					
LAMP-negative	0	58					
All (n = 76)			100 (94.1-100)	100 (78.2-100)	100 (78.2-100)	100 (94.1-100)	100 (76/76) (95.3-100)
LAMP-positive	15	0					
LAMP-negative	0	61					

In the Philippines (n = 76), most participants had a Ct value ≤30 (96.1%, n = 73). The accuracy for Ct values ≤30, ≤32 (97.4%, n = 74), and ≤35 (97.4%, n = 74) was 100% for all subjects, with 95% CIs of 95.1-100, 95.1-100, and 95.1-100, respectively. In addition, the accuracy for the number of days from symptom onset to the LAMP procedure was 100% for ≤2 (78.9%, n = 60), ≤3 (94.7%, n = 72), and ≤4 (96.1%, n = 73) days, with 95% CIs of 94.0-100, 95.0-100, and 95.1-100, respectively.

## Discussion

In this study, we explored an alternative diagnostic method for COVID-19 in Indonesia and the Philippines, where limited medical and technical resources make the gold-standard RT-PCR method less feasible. We focused on the LAMP method and assessed its efficacy. The LAMP and RT-PCR methods yielded equivalent results in 243 of 256 subjects (94.9%), indicating that LAMP is as effective as RT-PCR.

We assessed accuracy by stratifying by Ct values. For Ct values, the Centers for Disease Control and Prevention (CDC) and the Infectious Diseases Society of America (IDSA) cite 28-32 as the cut-off for successful viral culture [17]. This is because fragments, rather than the entire 30,000-bp SARS-CoV-2 genome, begin to be detected at these Ct values. In previous studies, many researchers have calculated the sensitivity and specificity of the LAMP method using Ct values of 30-35 as a cut-off [18-21]. Considering previous studies, our study set the cut-off for the Ct values at 30, 32, and 35. As a result, across all positive subjects in both countries, the accuracy of the LAMP and RT-PCR methods was 96.8% when the Ct value was ≤35. Furthermore, for Ct values ≤32 and ≤30, the accuracy was 99.6% and 100%, respectively. Therefore, the accuracy, viewed by Ct value, was high for Ct values ≤30 and ≤32. These results were consistent with those of previous studies.

In addition, we examined the consistency of the LAMP and RT-PCR results regarding the number of days from symptom onset to LAMP. The median number of days from symptom onset to the LAMP procedure was 1.5 days (IQR: 1-3), and within four days of symptom onset, 89.5% of the subjects were enrolled. Previous reports have shown that SARS-CoV-2 can be cultured for approximately two weeks after symptom onset [22,23]. Although further investigation is needed for the period from day 5 to two weeks after onset, we have shown that the LAMP and RT-PCR methods have high accuracy if the onset is within four days.

In Indonesia, 151 of 180 subjects (83.9%) had a Ct value ≤30, and 172 subjects (95.6%) were enrolled within one week of symptom onset. On the other hand, in the Philippines, 73 of 76 subjects (96.1%) had a Ct value ≤30, and all subjects (100%) were enrolled within one week of symptom onset. Therefore, the better results achieved in the Philippines may be due to a shorter time since symptom onset and a higher proportion of subjects with low Ct values.

Despite the high specificity and PPV, the NPV in the Indonesian cohort was 83.3%. In low-prevalence settings, NPV is a significant indicator for ruling out infection. An NPV of 83.3% means that 16.7% of truly infected individuals would be classified as false-negative cases. However, we reviewed Indonesian cases where LAMP and RT-PCR results did not match and concluded that the LAMP method may have limitations for identifying cases with high Ct values and low viral loads, whereas it can be useful for cases with high viral loads.

The number of subjects enrolled in this study was sufficient to support the use of the LAMP method to diagnose COVID-19 and met the EUL criteria for at least 100 cases per group, as recognized by the WHO [16]. In previous LAMP method studies in Japan [10,11,15,24], the PPV was 88.0%-100%, the NPV was 55.6%-100%, the sensitivity was 78.9%-100%, and the specificity was 97.6%-100%. We show comparable results in Indonesia and the Philippines.

An advantage of the LAMP method is faster detection compared to the RT-PCR method (35 min vs. 90 min). It comes in lyophilized form, eliminating the need for cold-chain logistics and providing an alternative solution for rapid COVID-19 detection. In addition, this study was conducted at three facilities in the urban areas of Indonesia and the Philippines that were well equipped to conduct LAMP and RT-PCR methods; however, both countries have many islands in their territories, and many areas have limited access to medical care. The usefulness of the LAMP method in such situations has been reported in previous studies on the diagnosis of tuberculosis and malaria [25-27]. Furthermore, the LAMP method is more cost-effective than RT-PCR, with the cost of LAMP testing estimated to be approximately 60% of that of RT-PCR per test, making it a more feasible diagnostic option in resource-limited settings [28,29]. As this study showed that the LAMP method has sufficient test accuracy for diagnosing COVID-19, it is expected that the LAMP method could play an active role in diagnosing COVID-19 even in remote areas with insufficient access to medical care and electricity infrastructure in island countries such as Indonesia and the Philippines.

Limitations

This study was conducted at one facility in the Philippines and two in Indonesia. The testing sites in this study were located in the city center, where access to health services was relatively easy. Therefore, it would be valuable to conduct further investigations at testing sites that better reflect the conditions in low- and middle-income countries or in areas with limited access to healthcare. Considering the high overall RT-PCR positivity rate of 50.8% in this study, there is a possibility that this result does not reflect clinical or community screening settings. There could be oversampling of severely symptomatic cases, indicating a spectrum bias. The high sensitivity (90%) and PPV (100%) reported here may not be generalizable to actual screening settings with low disease prevalence. Furthermore, there was an imbalance in the sample size and positivity rate between the Indonesian cohort (n = 180, 63.9% positive) and the Philippine cohort (n = 76, 19.7% positive). This imbalance may be attributable to the higher proportion of hospitalized patients in the Indonesian cohort, including more severe cases. Consequently, this difference may have introduced bias into the overall performance metrics and may limit the balance of the study as a multinational investigation. The Philippine subgroup had insufficient statistical power because of the small sample size, with 15 positive cases. Consequently, the reported 100% sensitivity and specificity in this subgroup may be statistically unreliable, as indicated by the wide CIs (e.g., sensitivity: 78.2%-100%).

## Conclusions

The high accuracy between the LAMP and RT-PCR methods indicates that the LAMP method could detect COVID-19 with the same level of accuracy as RT-PCR. Although the LAMP method is not currently listed under the WHO Emergency Use Listing, obtaining such international recognition in the future may facilitate its inclusion in national guidelines, particularly in low- and middle-income countries. This simple and effective diagnostic method may be especially beneficial in settings with limited access to healthcare.
